# Communicating blood test results in primary care: a mixed-methods systematic review

**DOI:** 10.3399/BJGP.2024.0338

**Published:** 2025-02-25

**Authors:** Helen Nankervis, Alyson L Huntley, Penny Whiting, William Hamilton, Hardeep Singh, Sarah Dawson, Rachel O’Donnell, Jane Sprackman, Anna Ferguson Montague, Jessica Watson

**Affiliations:** Population Health Sciences, Bristol Medical School, University of Bristol, Bristol.; Population Health Sciences, Bristol Medical School, University of Bristol, Bristol.; Population Health Sciences, Bristol Medical School, University of Bristol, Bristol.; University of Exeter, Exeter, UK.; Center for Innovations in Quality, Effectiveness and Safety (IQuESt), Michael E DeBakey Veterans Affairs Medical Center and Baylor College of Medicine, Houston, Texas, US.; Population Health Sciences, Bristol Medical School, University of Bristol, Bristol, UK.; Population Health Sciences, Bristol Medical School, University of Bristol, Bristol, UK.; Population Health Sciences, Bristol Medical School, University of Bristol, Bristol, UK.; Population Health Sciences, Bristol Medical School, University of Bristol, Bristol, UK.; Population Health Sciences, Bristol Medical School, University of Bristol, Bristol, UK.

**Keywords:** communication, haematologic tests, primary health care, systematic review

## Abstract

**Background:**

Online records access, including test results, was rolled out as part of changes to the GP contract in England in 2023. Blood test result communication is important for patient-centred care, patient safety, and primary care workload. Evidence is needed to ensure that test results are communicated safely and efficiently to patients in primary care.

**Aim:**

To summarise existing evidence for blood test result communication between primary care providers and their patients and carers.

**Design and setting:**

A mixed-methods systematic review was undertaken.

**Method:**

MEDLINE, Embase, PsycInfo (Ovid), CINAHL (EBSCOhost), and the Cochrane Library were searched from January 2013–September 2023. Qualitative or quantitative studies that provided information on the communication of blood test results by primary care staff to adult patients and carers were eligible for inclusion.

**Results:**

There were 71 included studies, including 10 experimental studies and no randomised controlled trials. Study quality was mostly poor and risk of bias was high, partly owing to a lack of reported information. The studies found that patients want more information about their blood test results, particularly in terms of ‘what next’, and prefer results to be provided quickly. Electronic methods, such as online access or text messages, were generally well accepted but not by everyone, and not for all results. Clinicians’ opinions were mixed as to whether online direct release of test results to patients was beneficial or could cause problems, such as increased patient anxiety and increased workload.

**Conclusion:**

A range of evidence has been identified on patient and clinician preferences, and barriers and facilitators to test communication, which is particularly important in the current NHS context of a move towards patient online access.

## Introduction

Around 14 tests are conducted per person in England and Wales annually.^1^ Evidence is needed to ensure that these results are communicated safely and efficiently to patients in primary care. Research to improve blood test communication is highly topical; since November 2023 all general practices in England have been required to provide adult patients with access to their full primary care record, including blood test results, through online accounts such as the NHS App.^2^ NHS case studies have suggested that improving online access to blood test results could reduce telephone calls to general practices to discuss results.^3^ However, the opposite is also possible, with primary care staff reporting concerns that viewing results online could lead to an increase in patient queries.^4^ The James Lind Alliance identified the need to provide information in patient medical records in a way that improves safety and quality of care as a priority.^5^ However, currently it is unclear how test results viewed in the medical record via the NHS App are perceived by patients. Moreover, not all patients will be able to access their test results online, so it is important to understand the evidence around alternative methods of test communication including telephone, face to face, and text message.

Test result communication is also important for patient safety, as communication may include instructions for repeating the test, follow-up tests or procedures, or starting treatment. The World Health Organization has identified that test result follow-up rates are suboptimal, leading to serious lapses in care.^6^ Studies quantifying failures in test result follow-up have been systematically reviewed, with between 6.8% and 61.9% of laboratory tests reportedly not followed up in US settings, with no relevant UK research identified.^7^

Safe and efficient systems for test result communication are therefore important for primary care workload, patient safety, and patient-centred care. The aim of this study was to summarise existing evidence for blood test result communication between primary care providers (for example, GPs, nurses, reception staff) and their patients and carers. The objectives were to review the benefits and potential harms of interventions, the needs and preferences of patients, clinicians, and healthcare staff, and the barriers and facilitators to blood test result communication.

**Table:** How this fits in

Communication of test result is important for patient safety, patient-centred care, and clinician workload. This systematic review included 71 studies of test communication in primary care — 41 quantitative, 19 qualitative, and 11 mixed-methods studies — with no randomised controlled trials identified. Patients want more information about their test results, particularly in terms of ‘what next’. Online access to test results is generally well accepted by patients, but reliance on electronic communication methods alone is not sufficient to meet the needs of different patients and different types of test result.

## Method

The study protocol was registered on PROSPERO (registration number CRD42023427433) and published.^8^ The review is reported according to the Preferred Reporting Items for Systematic reviews and Meta-Analyses (PRISMA) guidelines.^9^

### Search strategy

MEDLINE (Ovid), Embase (Ovid), PsycInfo (Ovid), CINAHL (EBSCOhost), and the Cochrane Library were searched from January 2013–September 2023, combining terms for communication (including ‘provider’, ‘nurse’, ‘doctor’, ‘communicat*’), patient access to records (including ‘access’, ‘patient’, ‘consumer’, ‘record[s]’), and test results (including ‘blood’ test[s]’, ‘result[s]’). Grey literature was also searched, including NHS websites, the reference lists of eligible full texts were hand-searched, and experts in the field were contacted. The search was restricted to the past 10 years to keep the review relevant, as communication knowledge, interventions, and technologies develop rapidly. The searches were optimised for each database via an iterative process (Supplementary Table S1).

### Study selection

Search results were exported to EndNote™ (version 20) and deduplicated before screening. Two reviewers independently screened titles and abstracts using Rayyan.^10^ Full copies of all reports considered potentially relevant were obtained and two reviewers independently assessed these for inclusion. Any disagreements were resolved by consensus or discussion with a third reviewer.

### Data extraction

One reviewer extracted study data using a piloted form, which was iteratively adapted. Data extraction was checked in detail by a second reviewer. Study authors were contacted for clarification, where necessary. Information including country, mode of communication, and types of tests studied were extracted (Supplementary Table S2).

### Quality assessment of included studies

Randomised controlled trials were assessed using the Risk of Bias 2 (RoB 2) tool,^11^ non-randomised studies of interventions using the Risk Of Bias In Non-randomised Studies — of Interventions (ROBINS-I) tool,^12^ non-randomised studies of exposure using the Risk Of Bias In Non-randomised Studies — of Exposures (ROBINS-E) tool,^13^ and cross-sectional studies using the Appraisal Tool for Cross-Sectional Studies (AXIS) quality assessment tool.^14^ Qualitative studies and the qualitative component of mixed-methods studies were assessed using the Joanna Briggs Institute (JBI) tool for qualitative studies.^15^

### Eligibility criteria

Primary studies of any design that provided information on the communication of blood test results by primary care staff (for example, doctors, nurses, physiotherapists, receptionists) and other providers (for example, primary care practices, primary care networks [PCNs], medical health insurance providers) to adult patients and carers were eligible for inclusion.

As ‘primary care’ varies across the world and has no single agreed definition,^16^ the authors defined primary care broadly as including all methods of care except those on emergency, urgent or acute care, or where the participants were inpatients.

**Figure 1.**
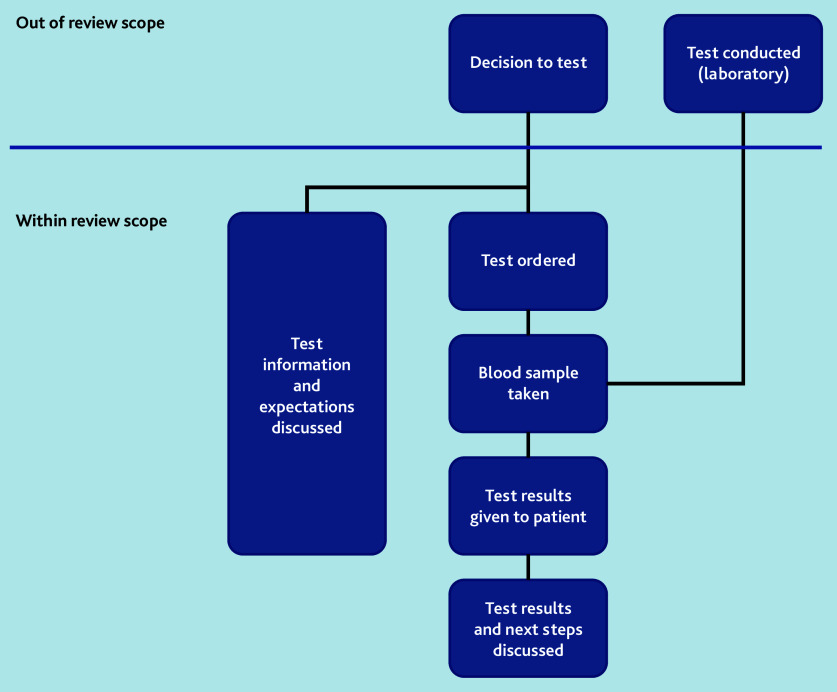
Scope of review.

The authors defined ‘communication of blood test results’ as any communication from the time of agreeing to order a test onwards, including what to expect from the results, when to expect the results, conveying the test results, how to interpret the results, and ensuring understanding of the next steps (Figure 1). This included the systems within primary care that aim to ensure that communication of blood test results to patients and carers takes place. Studies were also included where artificially generated data or hypothetical scenarios were used. Case studies, studies of point-of-care or genetic tests, studies exclusively in children, or communication of test results from laboratories to primary care were excluded.

### Data synthesis and integration

The review synthesis was conducted following JBI guidance for mixed-methods systematic reviews using a segregated convergent approach.^17^ For synthesis of the qualitative data, the meta-aggregative approach was used.^15^^,^^18^ Meta-analyses of quantitative data were not possible as there was a lack of homogeneous data on test result communication interventions. The findings from qualitative and quantitative data were synthesised separately, using narrative synthesis methods and the Synthesis Without Meta-analysis (SWiM) guidance.^19^ The syntheses of the qualitative and quantitative data were then combined and configured using juxtaposition and organised using the review objectives to create the convergent synthesis.

### Patient and public involvement

Two authors of this review are patient and public involvement (PPI) participants. A PPI group provided input into the design of the protocol and were consulted on the review conduct and reporting. The PPI input highlighted aspects of blood test result communication that were important to patients and carers. The points raised by the PPI group were used to guide the synthesis of the results, including highlighting when studies from a particular country (such as the US) were the majority of the evidence for a point and discussing the evidence in relation to ‘normal’ and ‘abnormal’ test results. Where evidence important to PPI participants was missing, these gaps were highlighted, such as drawing attention to whether carers were included in the evidence base.

## Results

### Summary of included studies

**Figure 2.**
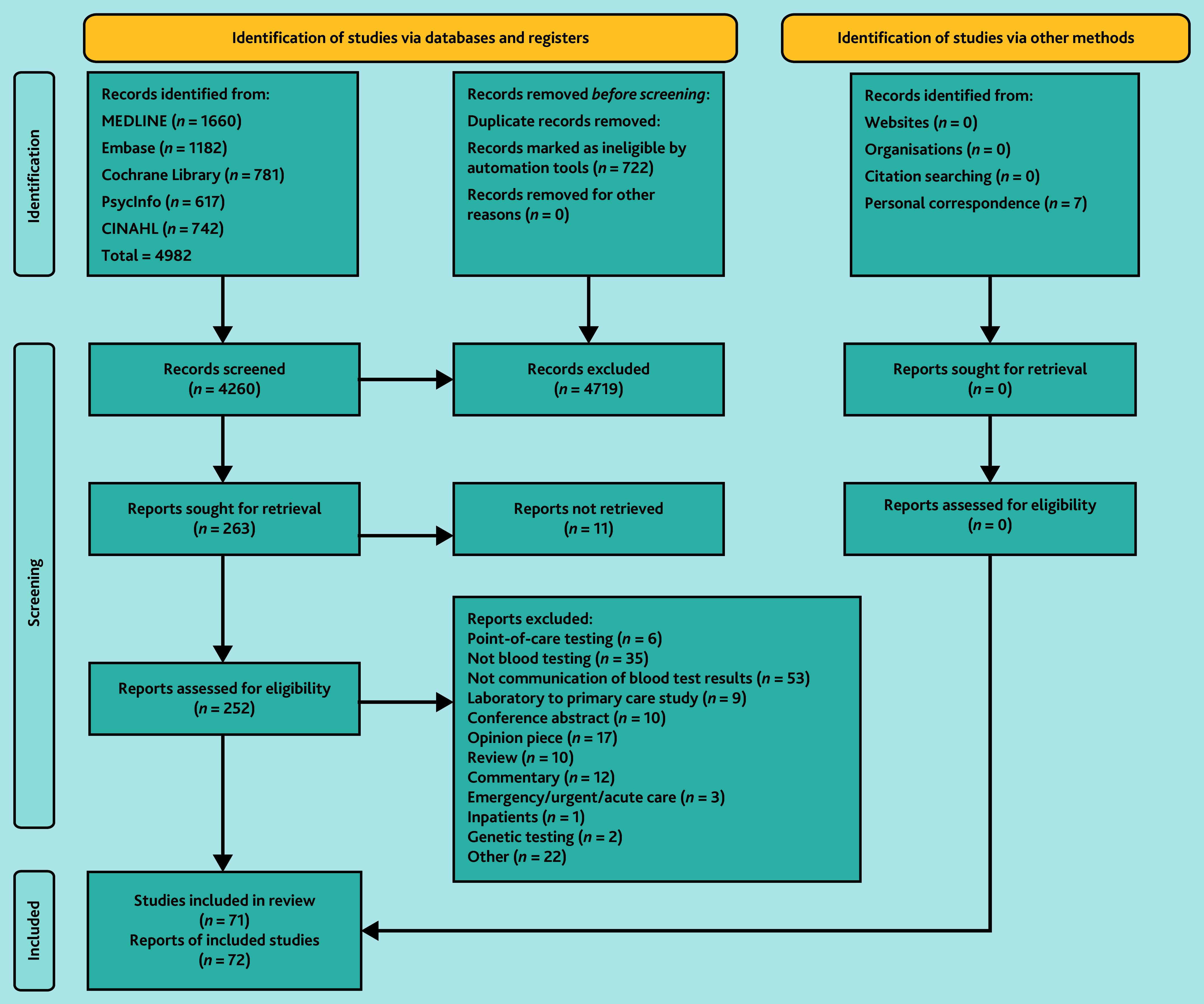
Preferred Reporting Items for Systematic reviews and Meta-Analyses (PRISMA) flowchart.

The searches identified 4982 records of which 263 records were sought for retrieval (Figure 2). There were 71 included studies (72 reports), of which 41 were quantitative, 19 were qualitative, and 11 were mixed-methods studies (Table 1). There were few experimental studies: one controlled trial, three uncontrolled pre–post studies, six randomised factorial studies, and no randomised controlled trials (Supplementary Tables S3–S5).

**Table 1.** Characteristics of included studies

**Feature**	**Category**	**Number of studies**
Type of study	Qualitative	19
	Quantitative	41
	Mixed methods	11

Study design	Randomised controlled trial	0
	Controlled experimental study	1
	Randomised factoral study	6
	Uncontrolled pre–post study	3
	Cohort study	1
	Cross-sectional study	35
	Unclear or other observational study design	28

Setting	UK	10
	US	30
	Rest of the world, multinational, or unclear	32

Sample size (individuals or organisations such as general practices)	1–100	32
	101–1000	21
	>1001	17
	Unclear	2

Participants	Patients or the general public only	42
	Clinicians and/or healthcare staff only	13
	Patients, clinicians, and/or healthcare staff	16
	Stakeholder groups	1

Mode of test communication	Online (including patient portals and electronic health records)	29

### Quality of included studies

#### Qualitative

Using the JBI tool, three quality indicators (Q6, 7, 8) were consistently not reported: no studies provided a cultural or theoretical positioning of the authors (Q6); none provided any description of their influence on the studies (Q7); and 22/28 studies did not adequately report the participants’ voices, with most offering only one quote to support a finding (Q8) (Supplementary Table S6).

#### Quantitative

Cross-sectional studies, assessed using the AXIS tool, had clear aims (Q1) 39/40 and an appropriate design (Q2) 37/40. The population of interest (Q4) was reported in 27/40 but the number of studies where the selection process was likely to lead to a representative sample of the population was low 8/40 (Q6), and the sample sizes were not justified (Q3) 39/40. Response rates raised concerns about non-response bias in 20/40 studies (Q13). Non-responders were addressed in 2/40 (Q7) and information about non-responders was described in 1/40 studies (Q14). The outcomes were appropriate for 33/40 studies (Q8); however, only 10/40 reported using previously piloted or validated outcome measures (Q9) (Supplementary Table S7).

The risk of bias was high for the six randomised factorial studies,^20^^–^^25^ which randomised patients to groups in which two or more variables, such as display format and test result normalcy, were assessed, and one controlled experimental study.^26^ The risk of bias was critical for two uncontrolled pre–post studies^27^^,^^28^ and serious for another.^29^ There was a high risk of bias for the one cohort study^30^ (Supplementary Table S8).

### Convergent synthesis

Table 2 summarises the convergent synthesis, which was configured according to the review objectives. Synthesis of the qualitative and quantitative data are shown in Supplementary Tables S9–S11.

**Table 2.** Summary of convergent synthesis results

**Review objective**	**Sub-categories**	**Included studies (*n*)**			**Summary of key findings**

		**Quantitative**	**Qualitative**	**Mixed methods**	
1. What interventions can be used to improve communication of blood test results to patients and carer in primary care?		3			No RCTs identified.
					No evidence of benefit for a question prompt list^29^ or for a standardised letter for test communication.^27^ Weak evidence of increased proportion of reduction in workload using enhanced automation to sort and flag critical abnormal results.^26^

2. Patients’ needs and preferences	Mode of communication preferences	14	5	3	Online portals were generally acceptable to patients,^31^^–^^36^ but preferences vary between patient populations^31^^,^^37^ and for sensitive or abnormal results.^31^^,^^32^^,^^36^^,^^40^^–^^42^ Text messages are generally acceptable, particularly for normal results.^28^^,^^35^^,^^45^^–^^47^ Some patients prefer telephone or face-to-face communication,^50^ particularly for sensitive or abnormal results.^45^
	Information needs and preferences Preferences for timing of test communication Preferences for level of access to results	5	4	4	Patients often needed and wanted to know more about the test results, particularly in terms of ‘what next’, including lifestyle changes and treatment options.^34^^,^^40^^,^^52^^–^^54^
		4	4	3	Patients preferred as little waiting time as possible; ‘delays’ were associated with frustration, anxiety, and avoidance.^31^^,^^32^^,^^35^^,^^36^^,^^38^^,^^41^^,^^51^^,^^57^
		4	3	2	Patients preferred to access test results easily, securely, at any time,^34^^–^^36^^,^^51^ with any level of ability,^36^^,^^53^ and to view as much or as little information as they wanted.^53^

3. Clinicians’ needs and preferences	Communicating result information to patients	3	2	1	US doctors were prepared to have normal test results released automatically to patients.^61^ There was a mix of opinions on whether providing further information about the tests would be beneficial.^47^
	Mode and timing of communication	3	4	2	Clinicians preferred interactive communication methods for more impactful results^33^^,^^35^^,^^45^^,^^62^^,^^64^ and passive methods where the results were normal.^33^^,^^45^^,^^62^ Clinicians were concerned that direct online release of results created patient anxiety and clinician workload.^65^
	Communication via healthcare staff	2			Unclear communication could have negative impact on staff, particularly receptionists,^66^ and training may help to improve this.^47^

4. Barriers and facilitators	Practical issues (that is, cost, travel, time spent)	5	7	2	Practical considerations (for example, cost, incorrect details, waiting) were reported as barriers to result communication.^35^^,^^46^^,^^51^^,^^66^^–^^71^ Literacy and numeracy may affect patient ability to interpret test results.^23^^,^^54^ Reference ranges and medical terminology confused patients.^23^^,^^38^^,^^39^^,^^60^
	Responsibility for test result communication	1	7	1	Lack of clarity around responsibility to follow-up test results had implications for patient safety.^47^^,^^51^^,^^57^^,^^62^^,^^66^ Clinicians often took responsibility for test communication where the patient was vulnerable, or results were likely abnormal and/or impactful.^64^^,^^72^
	Workarounds		2	3	Electronic or paper-based manual steps used in addition to standard process (for example, paper lists) can cause frustration, duplication, and increased workload.^64^^,^^66^^,^^67^^,^^73^^,^^74^ These were used to cope with poor organisation and communication of data,^73^ and to avoid safety issues.^64^
	Alerts and notifications	2	2		The number of electronic alert notifications was perceived as too high.^27^^,^^74^^,^^75^ Nevertheless, they were believed to reduce risks of failure to communicate abnormal results.^68^
	Physician clinician behaviour	0	2	3	Negative emotional reactions,^66^^,^^76^ providing unclear test result information,^66^ or ordering sensitive tests without informing the patient,^69^ were reported as barriers to communication.
	Display format and content of results	9	4	2	Presentation of test results in graphical or tabular format, use of goal or reference ranges,^77^ and additional information in video or verbal formats helped patients understand the results.^21^^,^^22^^,^^25^^.^^35^^,^^40^^,^^53^^,^^63^

*RCT = randomised controlled trial.*

### Objective 1: What interventions can be used to improve communication of blood test results to patients and carers in primary care?

No randomised controlled trials of interventions were found. There was no evidence of benefit for a question prompt list^29^ or for a standardised letter for test communication^27^ from two very small, poor-quality before-and-after studies. There was weak evidence from a non-randomised controlled study of increased performance (percentage of new or previously identified abnormal test results appropriately acted on by US doctors) and reduction in workload for using an electronic health record with enhanced automation to help sort and flag critical abnormal results. The trial was assessed as having a high risk of bias.^26^

### Objective 2: Patients’ needs and preferences

#### Mode of communication preferences (22 studies)

##### Portals (online access)

Receiving test results through patient portals (via an online interface) was viewed as acceptable or preferred, particularly if patients had used a portal before^31^^,^^32^ or if results were normal or routine.^31^^,^^33^ Perceived benefits of online access included being able to share results with multiple health providers, reducing consultations, and being more informed before consultations.^34^^–^^36^ Preferences were mixed, even in the same populations, and not all patients preferred online communication.^31^^,^^37^ Patients in Sweden wanted to receive results via letter in addition to online.^38^^,^^39^ Preference for online access when results were ‘bad news’ or sensitive was mixed,^36^^,^^40^ with some patients reporting that it was unacceptable^41^^,^^42^ unless this resulted in a delay,^31^ while some found it acceptable or preferred it.^32^ Patients wanted more information about electronic medical records, and they were not always clear about how they worked.^43^ In the US military, older patients but not younger patients preferred online access.^44^

##### Text or short message service (SMS) messaging

Communication of test results by text message was acceptable, particularly for normal results,^28^^,^^35^^,^^45^^–^^47^ even where the result may be sensitive (for example, HIV tests);^46^ however, this varied^35^^,^^48^^,^^49^ and was lower for older people,^28^^,^^45^ those with multiple health conditions,^45^ or if the results were abnormal.^35^^,^^45^ Trust in the clinician raised willingness to receive results by text in patients with cancer.^49^

##### Telephone or face to face

Some patients expressed a preference for test results to be communicated directly via telephone or in a consultation,^50^ particularly for sensitive, problematic, or abnormal results.^45^ Specialist doctors were preferred to primary care doctors for providing cancer test results,^42^ and doctors preferred to nurses for results that required changes in medication dose.^33^ Patients expressed concern about results being communicated by non-clinically trained staff, such as receptionists, for all but routine and minor tests.^35^^,^^51^

#### Information needs and preferences (13 studies)

Patients often needed and wanted to know more about the test results than was provided to them, particularly in terms of ‘what next’, including lifestyle changes and personalised treatment options.^34^^,^^40^^,^^52^^–^^55^ They found the test result information provided through a portal inadequate.^31^^,^^41^ Patients sought additional information by reading information provided with test results, contacting doctors, family, and friends, and the internet. This was more likely if results were abnormal and if additional information from the clinician was not available.^24^^,^^32^^,^^52^^,^^53^^,^^56^ Patients who were unsure if they needed follow-up after viewing their results online were more anxious than those who knew whether follow-up was needed.^43^ One UK study^57^ found patients prefer to have all their test results communicated to them, including ‘normal’ results. Some patients reported being unable to determine whether results were normal or abnormal and not understanding terms such as ‘negative’ and ‘non-reactive’.^53^

#### Preferences for timing of test communication (nine studies)

Patients preferred as little waiting time as possible, even when the results could be sensitive; ‘delays’ were associated with frustration, anxiety, and avoidance. Patient-portal users chose very short times (mostly 1 day) or immediate release of test results and tended to pick a shorter duration if they changed their mind about waiting time.^31^^,^^32^^,^^35^^,^^36^^,^^38^^,^^41^^,^^51^^,^^57^ Reasons were varied, including time to research implications of test results, processing bad news, and thinking about questions to ask. Some patients, including patients with cancer, would have preferred to have delayed results as it caused anxiety waiting to discuss them with a clinician. Higher health literacy was associated with a preference for shorter waiting times.^38^ Patients preferred close-ended timing information (for example, results will be available in 2 weeks); this did not vary for different tests.^58^

#### Preferences for level of access to results (nine studies)

Patients preferred to access test results easily, at any time,^34^^–^^36^^,^^51^ with any level of ability,^36^^,^^53^ and to view as much or as little information as they wanted.^53^ Patients preferred test results to be communicated confidentially^51^^,^^55^ and some wanted to know who would have access to them.^59^ Patients were comfortable with methods of communication with a high level of access control, including letters, password-protected websites, and personal voicemail or email, but were not always comfortable with fax, text message, and voicemail.^48^ Patients’ preferences were split on whether they should access test results at the same time as doctors and whether doctors should be allowed to withhold results until they had seen and discussed them.^60^

### Objective 3: Clinicians’ needs and preferences

#### Communicating result information to patients (six studies)

Doctors in the US and Australia were prepared to have normal test results released automatically to patients.^61^ Healthcare staff were frustrated with the time and resources to communicate normal test results,^57^ with text messaging seen as a time-saving mode of communication.^62^ Healthcare staff thought a leaflet with links to further information about tests would be beneficial for patients even if they received results online.^47^ There was concern that older patients would not adapt to changes in telephone communication such as a separate line for test results.^47^ There was a preference for colourful pictorial test result formats compared with tables or simple designs.^63^ Doctors had variable opinions about whether additional information on test result reports would benefit patients or their relationship with them owing to the accessibility of information via the internet and other sources.^55^

#### Mode and timing of communication (nine studies)

Clinicians preferred interactive communication, such as phone calls and return visits, where results required more input or would have more impact such as a change of medication or additional testing.^33^^,^^35^^,^^45^^,^^62^^,^^64^ Clinicians and healthcare staff preferred more passive methods of communication, such as asking patients to contact the provider, email, text messages, and online access, where the results were normal;^33^^,^^45^^,^^62^ however, there was also concern that communication of normal results was not necessary.^47^^,^^57^^,^^62^ Up-to-date contact information was important, particularly where the recipient’s identity could not be confirmed in real time such as email, phone voicemail, and text messages.^57^^,^^62^ Doctors with experience with using patient portals and direct release of test results were more comfortable with this than those unfamiliar with it.^61^ Doctors were concerned that direct online release of test results created additional anxiety for patients and extra workload for them.^65^ Communicating several test results to a patient, often spread out over time as they were not all received at once, created higher workload and stress for UK healthcare staff.^62^

#### Communication via healthcare staff (two studies)

Healthcare staff, particularly receptionists, were impacted by the quality and nature of test communication from clinicians to patients.^66^ They experienced frustration, anxiety, and pressure, owing to a lack of clarity and use of medical terms, which impacted communication of test results to patients.^66^ Communication of test results from doctor to healthcare staff and then to patients would benefit from clarity of communication and training.^47^

### Objective 4: Barriers and facilitators

#### Practical issues (14 studies)

Practical considerations,^46^ such as vacations,^67^ clinicians leaving,^67^ finding technology difficult,^68^ poor health or limited mobility,^51^^,^^69^ costs,^68^^,^^69^ time and distance for return trips,^51^^,^^69^ incorrect contact information or inability to contact patients,^66^^,^^68^^,^^70^^,^^71^ availability of appointments,^35^ and waiting on the phone for results,^51^ were reported as frustrating or potential barriers to communication, which were reported by mostly patients from the US and the UK.

Literacy and numeracy may affect patient behaviour and their ability to interpret test results.^23^ Patients with sufficient health literacy were interested in additional test information and resources.^54^ Patients were sometimes confused by information, including reference ranges and medical terminology.^23^^,^^38^^,^^39^^,^^60^ Telephone calls to primary care practices increased after normal tests were directly released online without doctor involvement.^65^

#### Responsibility for test result communication (nine studies)

There were different opinions and a lack of clarity among clinicians, healthcare staff, and patients around who had responsibility to follow-up test results, with implications for patient safety.^47^^,^^51^^,^^57^^,^^62^^,^^66^ Clinicians often took responsibility for test communication where they were concerned, or assessed that the patient was vulnerable, or the results were likely to be abnormal and/or impactful.^64^^,^^72^ Having no dedicated test management staff or inadequate staffing for a reliable point of contact was reported as a concern by US primary healthcare staff.^70^^,^^71^

#### Workarounds (five studies)

Workarounds are electronic or paper-based manual steps used in addition to standard process. Examples include keeping paper lists, or setting electronic reminders to ensure important results are followed up to avoid patient safety issues.^64^ These can cause frustration, duplication, and increased workload.^64^^,^^66^^,^^67^^,^^73^^,^^74^ Clinicians and healthcare staff used workarounds to cope with poor organisation and communication of data,^73^ and to avoid safety issues^64^ such as losing test results.^73^ Use of mechanisms to stop test results being missed was associated with a lower risk of test communication breakdown.^67^

#### Alerts and notifications (four studies)

The number of electronic health record-based alert notifications that clinicians receive was perceived as too high and the lack of separating the notifications based on urgency may have created a barrier in communication of test results.^27^^,^^74^^,^^75^ Nevertheless, having notifications about test results was believed to facilitate the testing process and reduce risks of failure to communicate abnormal results, which was important for patient safety.^68^

#### Clinician behaviour (five studies)

When communicating test results, negative emotional reactions,^66^^,^^76^ providing unclear test result information to healthcare staff,^66^ or ordering sensitive and impactful tests (for example, HIV) without the patient initially requesting them^69^ may be barriers. These behaviours have been associated with non-collection of results,^69^ healthcare staff communication of results to patients being inhibited,^66^ and patients’ lack of satisfaction with the communication of results.^76^ Clinicians may present ‘bad’ test results as numbers alone to help them avoid making moral characterisations and balance the doctor–patient relationship.^78^

#### Display format and content of results (15 studies)

Presentation of test results in graphical or tabular format, use of goal or reference ranges,^77^ as well as additional information in video or verbal formats helped patients achieve a better understanding of the results.^21^^,^^22^^,^^25^^,^^34^^,^^40^^,^^53^^,^^63^ This was effective when the results were ‘borderline’ (that is, slightly out of range) but did not necessarily make a difference when results were clearly out of range.^20^ When the general public were surveyed, medical phrases for blood test results seemed to be well understood; however, when discussing their views and experiences, patients believed they were a barrier to interpreting test results.^53^^,^^79^^,^^80^

## Discussion

### Summary

Existing evidence on test communication is heterogeneous, with a lack of interventional studies, no randomised controlled trials, and no evidence-based best-practice strategy for communication of blood test results. Patients want more information about their blood test results, particularly in terms of ‘what happens next’, and prefer test results to be provided quickly. Electronic methods, such as online direct access or text messages, were generally well accepted but not by everyone, and not for all results. Presentation of test results in a visual, graphical, or tabular format could help patients to achieve a better level of understanding of tests. Clinicians’ opinions were mixed as to whether more information and direct release of test results to patients without clinician input was beneficial or could cause problems such as increased workload. Barriers to test communication included time pressures and unclear processes, uncertainty about who was responsible for follow-up, as well as the burden of workarounds, electronic health record-based alerts, and notifications; overcoming these barriers is important for patient safety.

### Strengths and limitations

PPI participants, including two authors of the review, provided input into this review throughout its lifecycle, which ensured the findings highlight the issues that patients and carers want answered. The study sought to review blood test communication in the primary care setting; however, the definition of ‘primary care’ varies around the world. A strength is that all relevant evidence was included by covering all care settings that were not inpatients, urgent, or acute care. The disadvantage of this broad approach is that the review does not provide a focused response for a UK definition of primary care, although the authors believe that the findings are mostly generalisable. A narrow definition aligned to UK primary care would have found fewer studies, and probably been less relevant. The principles of good communication of test results are generalisable across the several care settings that were included.

Broad inclusion criteria were created using the authors’ knowledge of the research topic and the evidence base. The resulting heterogeneity and quality of reporting of the included studies meant that it was not possible to quantitatively synthesise the evidence. Creating a search around the broad criteria and the many different terms currently being used for test communication was challenging. In balancing the need for an achievable review with the sensitivity and specificity of the review, it is acknowledged that some studies may have been omitted.

### Comparison with existing literature

A review of adult patient access to electronic health records found that the effects were mostly uncertain.^81^ A review of different methods of communicating cancer screening results (letter, phone, and in-person) found that the most appropriate method of communication was not known owing to the lack of high-quality evidence and different patient preferences.^82^ One systematic review found that pictorial health information (not limited to test result communication) moderately improved patients’ knowledge and understanding, particularly among lower health literacy populations.^83^

### Implications for research and practice

The NHS in England is rolling out online access to blood test results. There has been a pushback against this move, with GPs fearing that patients will worry more, or find their GP records more confusing than helpful.^84^ However, evidence from countries such as the US, where patient portals have been studied for more than a decade, suggests these fears may be largely unfounded, with research showing online access is generally well accepted by patients. Various patient-centred tools for displaying test results in a visual or graphical way have been developed to improve patient understanding, which could be applicable to online portals in the UK such as the NHS App. Attention should be paid to the level of accompanying information provided by clinicians to ensure that comments are written in patient-friendly language, with patients particularly wanting more information about ‘what next’. There is a need for further evidence about the impact of the current shift towards online access to test results on primary care workload.

One clear message from this review is that reliance on the NHS App for test communication is not sufficient. A range of methods for test communication are needed to meet the needs of different patients, and for different test results. Practices should ensure they have clear protocols for communicating abnormal or impactful blood test results to ensure patient safety.

There are aspects of test communication that the evidence has not addressed. There were no trials of interventions such as patient portals to assess the benefits and harms. There was little assessment of face-to-face communication, which is still a major mode of blood test result communication in primary care.

Patients who do not use online technology and those with barriers to accessing primary care are not adequately represented in the studies included in this review. Many studies selected their population via online portals or via online questionnaires. Further research is needed to explore the experiences and preferences of people at risk of digital exclusion, for example, older people, minority ethnic groups, people without English as a first language, and those with additional needs. This is important to ensure that the move to online test result communication does not increase health inequalities. No research was found about communication of blood tests to carers, which the study’s PPI representatives felt was an important and common scenario.

In conclusion, online access to test results is generally well accepted by patients, but reliance on electronic communication methods alone is not sufficient to meet the needs of different patients and different types of test result. When accessing test results, by whatever method, patients need enough information to know what the results mean for their health, and what happens next. GPs should therefore ensure that comments added to blood test results are written in patient-friendly language. Practices should ensure they have clear protocols for communicating abnormal or sensitive results, and should explain methods of test communication to patients at the time of testing. Improving test result communication is important for patient-centred care, patient safety, and primary care workload.
